# Psychometric functions for temporal discrimination: Duration or log duration?

**DOI:** 10.3758/s13428-026-03075-0

**Published:** 2026-06-12

**Authors:** Miguel A. García-Pérez, Rocío Alcalá-Quintana

**Affiliations:** Departamento de Metodología, Facultad de Psicología, Universidad Complutense, Campus de Somosaguas, 28223 Madrid, Spain

**Keywords:** Duration discrimination, Psychometric function, Weber’s law, Fechner’s law

## Abstract

Duration discrimination data collected with single-presentation tasks (e.g., bisection or temporal generalization tasks) or dual-presentation tasks (e.g., greater–less or same–different tasks) are usually analyzed by fitting psychometric functions. The independent variable in these functions is test duration measured in seconds or milliseconds. In contrast, the independent variable is log stimulus magnitude in psychometric functions for discrimination in other sensory modalities, most often because of the applicability of Weber’s law. We report a study aimed at determining empirically whether duration discrimination data are best described by psychometric functions of duration or log duration. We first conducted a simulation study to identify the design (type of task, number of test durations, number of trials, etc.) with which the generating psychometric function (of duration or log duration) fit the data substantially better than the impostor function. Based on these results, we conducted an empirical study with the same–different task that adaptively administered 1,200 trials over 11 test durations around the standard duration. We collected 45 datasets and fitted both types of psychometric function in each case. By the loglikelihood-ratio statistic, psychometric functions of duration fitted the data better in only 7 of 45 cases (15.6%), in agreement with simulation results obtained when data were generated by psychometric functions of log duration. Analysis of data from 69 published papers (totaling 17,000+ psychometric functions) also indicated better fit of psychometric functions of log duration in the expected proportion given the typically small numbers of trials per function.

## Introduction

Weber’s law states that the smallest detectable increment ∆*S* in the physical magnitude *S* of a stimulus (also known as the *just noticeable difference* or the *difference limen*) is proportional to *S*, that is, ∆*S* = *k*_w_ × *S*, where *k*_w_ is known as the Weber fraction. Fechner’s law, in turn, states that the perceived magnitude ψ of a stimulus is proportional to the logarithm of its physical magnitude *S*, that is, ψ = *k*_f_ × log(*S*), where *k*_f_ is a scaling factor sometimes referred to as the Fechner constant. These laws have guided psychophysical research for more than 150 years (see Laming, 2010) and they have several implications, one of them affecting properties of the mathematical form of psychometric functions. A psychometric function describes how the probability of a certain type of judgment in a psychophysical task varies with stimulus magnitude. For instance, the psychometric function for temporal discrimination typically describes how the probability of judging that the duration of a test stimulus was longer than that of a reference stimulus increases with the presentation duration of the test stimulus. Weber’s and Fechner’s laws imply that stimulus magnitudes expressed in log units render psychometric functions that are slope-invariant. Thus, in visual contrast perception, the psychometric function is defined as a function of the logarithm of luminance contrast (e.g., Nachmias, 1981), and in auditory loudness perception the psychometric function is defined as a function of the logarithm of sound pressure level (e.g., Hall, 1981).

A remarkable exception to this principle arose in the area of time perception and, more specifically, in the study of discrimination of temporal duration (i.e., duration discrimination). In this area, perceived duration is almost invariably regarded as a linear function of actual duration, without any intervening logarithmic transformation (e.g., Lapid, 2008). The reasons for this decision are unclear, and the current prevalence of such a practice may simply reflect how an arguably unfortunate choice was perpetuated. In fact, Creelman’s (1962) theory of time discrimination posited that duration discrimination is based on the difference in the number of pulses that a counting mechanism receives during each of the temporal intervals to be compared. In his theory, the number of pulses generated over an interval of duration *T* is a Poisson random variable with mean λ*T*, not λlog(*T*), where λ is the Poisson rate parameter (see also Rammsayer & Ulrich, 2001). Thus, the variance of the number of pulses increases with *T*, which captures Weber’s law. Later on, scalar expectancy theory (SET; Gibbon, 1977) analogously posited that the perceived duration of an interval of duration *T* is a Gaussian random variable with mean and standard deviation proportional to *T*, not to log(*T*). This is compatible with Weber’s law, and one might argue that Fechner’s law does not necessarily apply in this context because time is not “perceived” by any sense organ, and definitely not by one that transduces the energy embedded in intensive physical magnitudes. Nevertheless, when Weber’s law holds, psychometric functions of log duration incorporate Fechner’s law and have a slope that is invariant with the magnitude of the standard duration, something that anecdotal data suggest holds in duration discrimination.

It should be noted that the issue of whether psychometric functions should be written out as functions of duration or log duration is unrelated to that of the strict validity of Weber’s law in time perception, that is, unrelated to the question of whether ∆*S* is proportional to *S* everywhere along the time continuum or, rather, ∆*S* holds a more complex relation to *S* (see Haigh et al., 2021; Bizo et al., 2006; Ulrich et al., 2023). The work described in this paper attempted instead to resolve empirically the question of whether duration discrimination data are more adequately described by psychometric functions of duration or log duration. This question is purely practical and unrelated to a discussion of whether the internal representation of time is linear or logarithmic. The two alternatives—namely, a linear representation with standard deviation proportional to time or a logarithmic representation with constant standard deviation—have been tested against data with mixed results that varied across methods used to investigate the issue but also across observers (see, e.g., Jozefowiez et al., 2018). Obtaining a clear answer to the question is hampered by lack of access to the internal representations themselves. Thus, psychophysical methods implement manipulations that elicit representations, and observers must perform tasks through which indirect access to the magnitude of the representations is obtained. However, temporal intervals presented during the experiment are mapped onto internal representations via the psychophysical function of interest and, analogously, internal representations are mapped back via the same function onto the actual times measured in the study. This inevitable two-way journey leaves the form of the mapping function concealed. Several attempts have been made to circumvent this problem (see, e.g., Ren et al., 2020; Zhou et al., 2024), but we will not discuss them here because they are not relevant to our goal.

With a focus on whether psychometric functions of duration or log duration better describe duration discrimination data, the competing functions are presented and compared in the next section, where the relevance, consequences, and implications of the distinction are discussed. Next, simulation studies are conducted to determine the conditions under which the two forms can be empirically distinguished, with an eye to determining the number of test durations at which the psychometric function must be sampled, the type of task that observers must be asked to perform, the sampling plan used to deploy trials, and the overall number of trials that should be administered. Subsequently, an empirical study is reported that uses optimal settings determined by the preceding simulations to collect data on discrimination of the temporal duration of an auditory stimulus, with the purpose of assessing which of the two competing psychometric functions fits the data better. We also report the results of analogous analyses of published datasets to check for similar patterns in extant data, although the original studies administered far fewer trials than our simulations indicate are necessary for a clear answer. In brief, (i) our simulation results show that the two forms of psychometric function are distinguishable upon administration of a relatively large but feasible number of trials, (ii) our empirical results show that data from a duration discrimination task with an auditory stimulus are better described by psychometric functions of log duration, and (iii) the analysis of published data generally aligns with our empirical results.

## Psychometric functions of duration versus log duration

This section describes the relevance of our research question along four fronts.

### Spread and symmetry

**Fig. 1:**
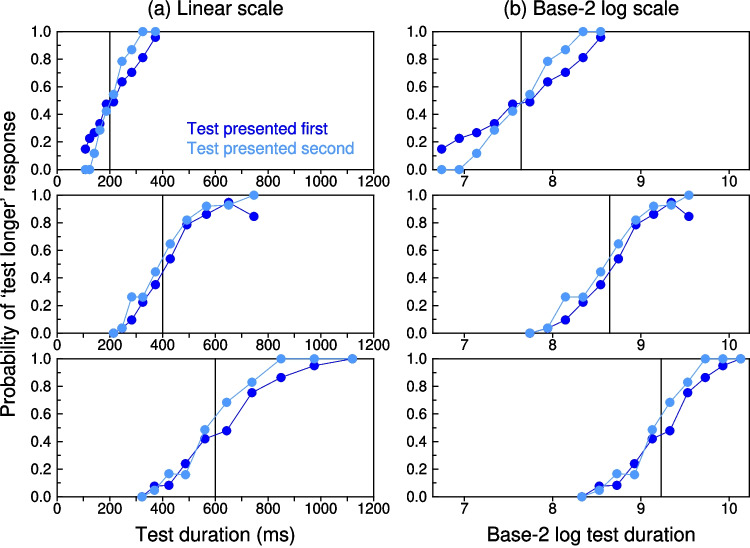
Duration discrimination data for three different standard durations (200 ms, 400 ms, and 600 ms; rows) plotted as a function of test duration (left column) or log_2_ test duration (right column). Data come from a single observer under the greater–less response format in a dual-presentation task in which the test duration was presented first (dark blue) or second (pale blue). The dashed vertical line in each panel indicates the standard duration

Figure 1 shows unpublished data (Alcalá-Quintana & García-Pérez, 2023) from one of six observers who performed a dual-presentation duration discrimination task. Data represent the proportion of trials in which the test duration was perceived to be longer than the standard duration, as a function of test duration and separately for trials in which the test was presented first or second. Durations were delivered visually by lighting an LED for the prescribed amount of time. Separate sessions collected data for five standard durations (from 200 ms to 600 ms in 100-ms steps), although Fig. 1 only displays results for three of them. Each session comprised several blocks that collectively deployed 720 trials over a set of 10 test durations adaptively chosen from a set that ranged from nearly half the standard duration to nearly twice the standard duration. Trials with each presentation order were randomly interwoven throughout each block. The two columns in Fig. 1 display the exact same data on a linear axis of test duration (left column) or a base-2 logarithmic axis of test duration (right column).

Three features of the data are worth mentioning. The first is that on the linear axis of Fig. 1a, the spread of the psychometric function broadens with increasing duration of the standard (Fig. 1a), whereas spread seems invariant with standard duration on a log axis (Fig. 1b). Secondly, the two strands of symbols in each panel follow slightly different paths, thus reflecting presentation order effects (Jamieson & Petrusic, 1975; see also Ellinghaus et al., 2024). The third feature is that on a linear axis (Fig. 1a), the strands of symbols to the left and to the right of the vertical line in each panel (indicating the standard duration) seem to follow paths with different slopes (steeper on the left and shallower on the right).^1^ In contrast, on a logarithmic axis (Fig. 1b), the slopes of the strands of symbols on either side of the vertical line appear more similar. The question that we address is whether a psychometric function of suitable form should be fitted to data expressed as duration or log duration (irrespective of how the data are plotted). Formally, and assuming that a logistic function is appropriate, the question is whether data collected with the greater–less response format should be fitted by

1a $$\Psi (x)=\frac{1}{1+\mathrm{e}\mathrm{x}\mathrm{p}\left[-b(x-a)\right]}$$

or by

1b $$\Psi \left(x\right)=\frac{1}{1+\mathrm{e}\mathrm{x}\mathrm{p}\left[-b\left(\mathrm{log}\left(x\right)-a\right)\right]},$$

where *x* is the test duration, and *a* and *b* are free parameters.

**Fig. 2:**
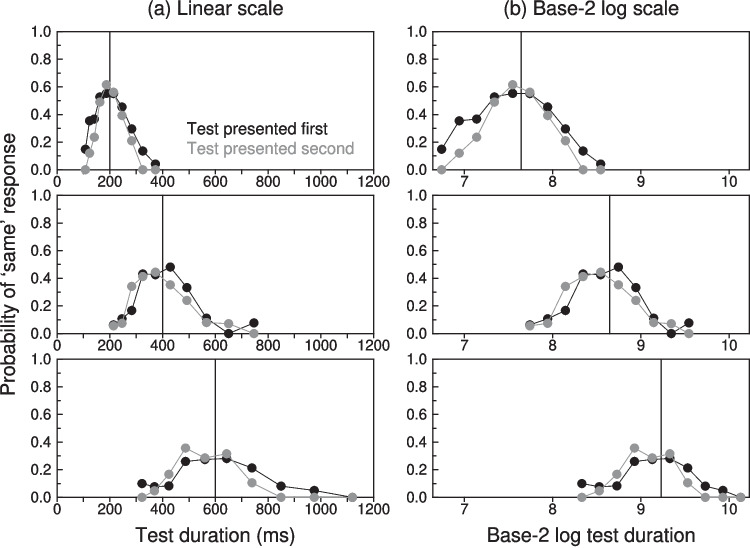
Duration discrimination data for the same standard durations and the same observer as in Fig. 1 but under the same–different task in which observers report whether the two presentation durations in each trial are equal or different. The test duration was presented first (black) in half of the trials and second (gray) in the other half. Data come from the same observer as in Fig. 1

Analogous characteristics are observed in data collected with the same–different response format where observers simply indicate whether the two presentation durations in each trial were equal or different. As seen in Fig. 2, in this case the data display a non-monotonic pattern that shows clear signs of asymmetry when plotted on a linear axis (Fig. 2a) and signs of symmetry when plotted on a logarithmic axis (Fig. 2b). The same pattern is observed in the single-presentation variant known as the temporal generalization task (see Wearden, 1992).

### Difference limen

**Fig. 3:**
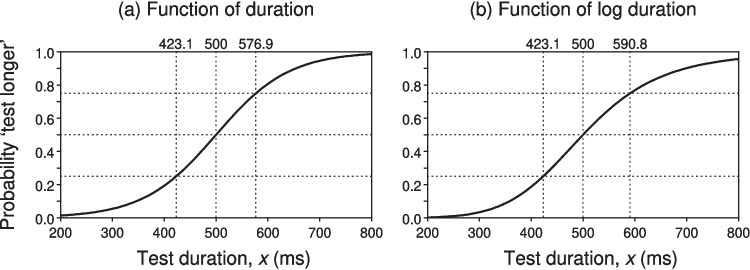
Differences between a logistic psychometric function given by Eq. 1a with *a* = 500 and *b* = 0.014 (left panel) and a logistic psychometric function given by Eq. 1b with *a* = 6.215 and *b* = 6.582 (right panel). The horizontal axis uses a linear scale in both cases to ease the comparison. Parameter values for each function were selected so that in both cases the 50% point on the curve lies at a test duration of 500 ms (central vertical dashed line in each panel) and the 25% point on the curve lies at a test duration of 423.1 ms (left vertical dashed line in each panel). Because of the symmetry of the curve in the left panel and the asymmetry of the curve in the right panel, the 75% point on each curve lies at a different test duration: 576.9 ms in the left panel and 590.8 ms in the right panel

Figure 3 illustrates one of the consequences of using functions of duration or functions of log duration for estimating the difference limen (DL) with the greater–less response format. The DL has been defined and estimated in several ways, but to make it consistent with the notion of just noticeable difference at the core of Weber’s law, consider it defined as the distance between the 75% and the 50% points on the psychometric function. The functions in Fig. 3 are indistinguishable by eye, but DL = 76.9 ms in Fig. 3a, whereas DL = 90.8 ms in Fig. 3b. In other words, estimates of the DL are larger by Eq. 1b than they are by Eq. 1a. Furthermore, an alternative method for estimating the DL consists of taking half the distance between the 75% and the 25% points on the psychometric function. With the symmetric function in Fig. 3a, the estimate is identical to that obtained above: (576.9–423.1)/2 = 76.9; with the asymmetric function in Fig. 3b, the estimate is (590.8–423.1)/2 = 83.85 and not 90.8 as it was above. The fact that estimates of the DL differ across functional forms of the psychometric function prompts the question of which of them is the “correct” one, which requires an answer to the question of what type of psychometric function describes the data more faithfully.

### Type B order effects

A further difference between the two formulations lies in their capability to account for Type B order effects whereby data and psychometric functions of duration plotted along a linear axis vary in slope across presentation orders (see Ulrich & Vorberg, 2009). The classical approach for dealing with Type B order effects consists of fitting separate psychometric functions to data collected with each order of presentation of test and standard, and with parameters that are allowed to differ across presentation orders (see, e.g., Bausenhart et al., 2012; García-Pérez & Alcalá-Quintana, 2011a; Ulrich & Vorberg, 2009). A congruent account requires an alternative framework in which the psychometric functions for each presentation order may have different functional expressions but they must share their parameters so that a single set of parameter values accounts for all the data. One such model will be described below, but for the current illustration it suffices to say that the psychometric functions Ψ_TF_ and Ψ_TS_ that hold for test-first and test-second presentations, respectively, under the greater–less response format in that model are given by

2a $${\Psi}_{\mathrm{TF}}(x)=\Phi \left(\frac{\delta -\beta ({x}_{\mathrm{s}}-x)}{\sqrt{2}}\right)$$

2b $${\Psi}_{\mathrm{TS}}(x)=\Phi \left(\frac{\beta (x-{x}_{s})-\delta }{\sqrt{2}}\right)$$

in the linear version or by

3a $${\Psi}_{\mathrm{TF}}(x)=\Phi \left(\frac{\delta -\beta \left(\mathrm{l}\mathrm{o}\mathrm{g}({x}_{s})-\mathrm{l}\mathrm{o}\mathrm{g}(x)\right)}{\sqrt{2}}\right)$$

3b $${\Psi}_{\mathrm{TS}}\left(x\right)=\Phi \left(\frac{\beta \left(\mathrm{log}\left(x\right)-\mathrm{log}\left({x}_{s}\right)\right)-\delta }{\sqrt{2}}\right),$$

in the log version, where Φ is the unit-normal cumulative distribution function, *x*_s_ is the standard duration, and β and δ are free parameters with the same values for both orders of presentation of test and standard durations.

**Fig. 4:**
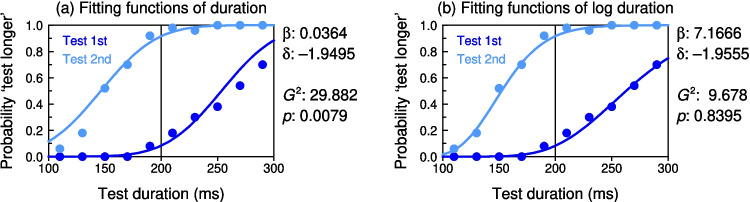
Psychometric functions of duration cannot accommodate data displaying Type B order effects (left panel) whereas psychometric functions of log duration can (right panel). The horizontal axis uses a linear scale in both cases to reflect conventional practices and to ease the comparison. Artificial data were generated from Eqs. 3 with β = 7.5 and δ = −2 for a standard duration *x*_s_ = 200 ms with 50 trials per test duration per presentation order. Values printed on the right of each panel are parameter estimates (top part) as well as the value of the log-likelihood ratio statistic *G*^2^ and its associated *p* value (bottom part)

The two panels of Fig. 4 show the same artificial data (symbols) generated by Eqs. 3 with β = 7.5 and δ = −2 for discrimination from a standard duration *x*_s_ = 200 ms using 50 trials per presentation order at each of 10 test durations. The panel on the left shows the results of fitting the linear model in Eqs. 2, which clearly do not do justice to the data. The fitted curves differ only by translation, with the result that the dark blue curve from Eq. 2a cannot capture the shallower path of the dark blue data points at long test durations, and the pale blue curve from Eq. 2b cannot capture the steeper path of the pale blue data points at short test durations. In contrast, fitting the log model in Eqs. 3 accommodates the paths of the data and captures the apparent Type B order effect without different slope parameters in each function.

Trivially, the best-fitting model is that which generated the data, but this is not the message that Fig. 4 carries. The point here is that determining whether empirical data should be described by psychometric functions of duration or by functions of log duration is necessary to investigate whether Type B order effects are an artifact of fitting duration discrimination data with the wrong psychometric function. We must stress that our use of a generative model to produce Fig. 4 has only an illustrative purpose. Our point is that some Type B order effects *may* arise if log duration is the relevant variable. Of course, Fig. 4 does not “explain” Type B order effects, and it also does not lend support to the model used to generate the figure. In fact, the model of Eqs. 3 cannot produce Type B effects without Type A effects. Capturing the process by which Type B order effects arise independently from Type A effects has proven elusive (see Ellinghaus et al., 2024), and we do not address this issue in the present paper. We also do not impose the equal-parameters assumption on any analyses presented in this paper.

### Empirical practices

**Fig. 5:**
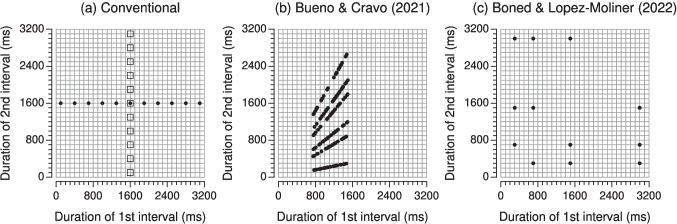
Sampling of the two-dimensional duration space in different studies. **a** Conventional sampling in which the standard duration (1,600 ms) is presented first and coupled with several test durations presented in the second interval (vertical strand of squares), or the standard duration is presented second and coupled with several test durations presented in the first interval (horizontal strand of circles). **b** Sampling points (circles) in the study of Bueno and Cravo (2021), where durations presented first and second differed by a scale factor. **c** Sampling points (circles) in the study of Boned and López-Moliner (2022), where durations presented first and second differed by linear increments.

The last consideration about the relevance of our research question bears on the justification for certain empirical practices. The most common scenario in duration discrimination studies involves the use of a given (fixed) standard duration and a number of repeat trials in which the standard duration is coupled with each of a set of test durations. According to whether the standard duration is presented first or second, the two-dimensional duration (2DD) space with dimensions “duration of the first interval” and “duration of the second interval” is sampled as illustrated in Fig. 5a. Thus, the psychometric function is defined along the (vertical or horizontal) line of test durations, and its functional form may be any of those mentioned above (i.e., Eqs. 1, 2, or 3 , among others) in their version for duration or log duration. A study might involve several standard durations, but they are all treated separately as just described. However, there are studies in which a standard duration does not exist in the conventional sense just mentioned. Yet, data in these cases are arranged and analyzed as if some standard existed, which implies assumptions about whether conventional psychometric functions differ only by translation or by both translation and dilation across standard durations. Next, we give two examples that illustrate the practical relevance of the research question that we address.

Bueno and Cravo (2021) reported a duration discrimination study with 180 trials per observer and condition. In each individual trial, the duration of the first interval was randomly sampled with equiprobability between 750 ms and 1,500 ms, and the duration of the second interval was *k* times the duration of the first interval, with *k* selected at random with equiprobability from the set {0.2, 0.6, 0.8, 1.2, 1.4, 1.8} under the constraint that each factor is used in exactly 30 trials. Obviously, this study does not have a standard duration as we know it, but rather, the 2DD space is sampled as shown in Fig. 5b for a particular realization of the randomly chosen pairs of first and second durations across the 30 trials with each of the six values for the factor *k*. Note that the sampling points thus created fall along separate lines, each with a slope equal to one of the possible values of *k*. Bueno and Cravo (2021) aggregated responses to trials falling on each of these six lines and fitted a psychometric function with *k* as the independent variable. In other words, the independent variable for this psychometric function is the factor that the duration of the second interval is to the duration of the first interval. Justification for this aggregation of data relies on the assumption that conventional psychometric functions for different standard durations have the same slope on a logarithmic axis, something that has never been established. Identical considerations hold for other studies lacking conventional standard durations and sampling the 2DD space analogously but regularly (instead of randomly) along lines with selected slopes (e.g., Riemer et al., 2022).

The study by Boned and López-Moliner (2022) arranged data in a different manner that also differs from the conventional approach. Across conditions in their study, trials paired three durations (300, 700, and 1,500 ms) or four durations (300, 700, 1,500, and 3,000 ms) with each other, excluding pairs with identical durations. Figure 5c shows how the 2DD space is sampled in the less sparse case that involves 12 pairs. In a conventional analysis, psychometric functions could have been fitted by regarding each individual duration as the standard presented first or second (i.e., using data on the same vertical or the same horizontal line in 2DD space), and this would have rendered eight conventional psychometric functions but with only three test durations for each standard duration. Now, the difference *d* between the duration of the second interval and the duration of the first interval can be computed to render 12 values (from 300–3,000 = −2,700 ms to 3,000–300 = 2,700 ms). Boned and López-Moliner fitted psychometric functions with *d* as the independent variable. In other words, the independent variable for these psychometric functions is the difference between the duration of the second interval and the duration of the first interval, something that is justifiable only if the psychometric functions for different standard durations have the same slope on a linear axis. This assumption is the exact opposite to that which justifies the analyses described in the preceding paragraph. Identical considerations hold for other studies lacking conventional standard durations and in which psychometric functions are regarded as functions of the difference between the duration of the two intervals in each trial (e.g., Lammers et al., 2023; Li, Wang, & Chen, 2022; Li, Wang, & Zaidel, 2026).

Clearly, either the aggregation procedure of Boned and López-Moliner (2022) or that of Bueno and Cravo (2021) is justifiable, but certainly not both. Our study is aimed at determining which of the two is supported by empirical data by determining whether psychometric functions of duration or log duration fit conventional duration discrimination data better. In search of an approach to find the answer, the next section uses simulation methods to determine how much data are required to distinguish whether they need to be fitted by psychometric functions of duration or by psychometric functions of log duration.

## Simulation study: Distinguishability of the two types of function

The guiding principle of this simulation study is that, given sufficiently informative data, the model that generated the data should fit them better than an alternative model. Our purpose here is to determine how much data need to be collected for them to be “sufficiently informative” in this sense. Thus, we separately generated data from psychometric functions of duration and from psychometric functions of log duration, and in both cases we fitted psychometric functions of duration and psychometric functions of log duration to the same data. We manipulated the number of test durations that probe psychometric functions, their spacing, the overall number of trials that were administered, and the deployment strategy (fixed or adaptive). We sought to determine the minimum number of trials needed for the *G*^2^ goodness-of-fit statistic to achieve consistently smaller values when the fitted model matches the generating model. The simulation study was conducted identically under greater–less tasks and same–different tasks. These two tasks are known to differ in the accuracy of the parameter estimates they provide (see Alcalá-Quintana & García-Pérez, 2013; García-Pérez & Alcalá-Quintana, 2017, 2020a), and they might additionally differ in their suitability for telling competing models apart. Because the ability to tell models apart should remain invariant with the order of presentation of test and standard durations in each trial, the simulation study generated data only for trials in which the test was presented second. This may seem an unnecessary simplification, because a simulation study does not incur any extra burden by doubling the number of trials to include cases in which the test is presented first. However, we seek to determine the optimal sampling plan for fitting individual psychometric functions in our subsequent empirical study.

### Design and procedure

The design of the main simulation study is a factorial 2 (generating model: linear or log) × 2 (fitted model: linear or log) × 2 (response format: greater–less or same–different) × 15 (overall number of trials: 100 to 1,500 in steps of 100) × 7 (number of test durations: 7 to 13) × 2 (spacing of test durations: linear or logarithmic) × 2 (deployment strategy: fixed or adaptive). We used a single standard duration *x*_s_ = 200 ms in all conditions. Variants of this main simulation study will be described below.

Data for conditions involving the greater–less response format were generated using Eq. 2b (for the linear model) or Eq. 3b (for the log model). These expressions arise from the indecision model in García-Pérez and Alcalá-Quintana (2017) for the degenerate case in which δ_2_ = δ_1_ = δ. Without loss of generality, we set β = 0.03 in the linear model and β = 6 in the log model for each of *n* = 5,000 replicates in each condition, with δ varying across replicates as a random variable uniformly distributed over [−0.75, 0.75] in either case. For conditions involving the same–different response format, data were also generated from the indecision model, but now with δ_2_ ≠ δ_1_. Although we only generated test-second data, the psychometric functions ϒ_TF_ and ϒ_TS_ that hold for test-first and test-second presentations under the same–different response format in this model are respectively given by

4a $${\Upsilon}_{\mathrm{TF}}(x)=\Phi \left(\frac{{\delta}_{2}-\beta ({x}_{\mathrm{s}}-x)}{\sqrt{2}}\right)-\Phi \left(\frac{{\delta}_{1}-\beta ({x}_{\mathrm{s}}-x)}{\sqrt{2}}\right)$$

4b $${\Upsilon}_{\mathrm{TS}}(x)=\Phi \left(\frac{\beta (x-{x}_{s})-{\delta}_{2}}{\sqrt{2}}\right)-\Phi \left(\frac{\beta (x-{x}_{s})-{\delta}_{1}}{\sqrt{2}}\right)$$

under the linear version and by

5a $${\Upsilon}_{\mathrm{TF}}(x)=\Phi \left(\frac{{\delta}_{2}-\beta \left(\mathrm{l}\mathrm{o}\mathrm{g}({x}_{s})-\mathrm{l}\mathrm{o}\mathrm{g}(x)\right)}{\sqrt{2}}\right)-\Phi \left(\frac{{\delta}_{1}-\beta \left(\mathrm{l}\mathrm{o}\mathrm{g}({x}_{s})-\mathrm{l}\mathrm{o}\mathrm{g}(x)\right)}{\sqrt{2}}\right)$$

5b $${\Upsilon}_{\mathrm{TS}}(x)=\Phi \left(\frac{\beta \left(\mathrm{l}\mathrm{o}\mathrm{g}(x)-\mathrm{l}\mathrm{o}\mathrm{g}({x}_{s})\right)-{\delta}_{2}}{\sqrt{2}}\right)-\Phi \left(\frac{\beta \left(\mathrm{l}\mathrm{o}\mathrm{g}(x)-\mathrm{l}\mathrm{o}\mathrm{g}({x}_{s})\right)-{\delta}_{1}}{\sqrt{2}}\right)$$

under the log version. Parameter β was set as described for the greater–less response format, whereas parameter δ_1_ was uniformly distributed over [−2.5, −1.0] and parameter δ_2_ was uniformly distributed over [1.0, 2.5] across replicates under both generating models. Same–different data were generated only from Eqs. 4b or 5b (i.e., for test-second presentations).

With log spacing, test durations ranged from nearly half the standard value (actually, ${x}_{\mathrm{s}}\times {2}^{-0.7}$) to nearly twice the standard value (actually, ${x}_{\mathrm{s}}\times {2}^{0.7}$) in evenly spaced binary log steps. Linearly spaced test durations covered the same range in evenly spaced linear steps. With the β parameters indicated above, this range of test durations probes the psychometric function appropriately without losing degrees of freedom for the *G*^2^ statistic due to small expected frequencies (for a thorough description of this problem, its consequences, and ways around it, see García-Pérez & Alcalá-Quintana, 2025).

Regarding the deployment strategy, the fixed form used the method of constant stimuli (MOCS) and displayed the same number of trials at each test duration. When the overall number of trials was not divisible by the number of test durations in the sampling plan, the remainder was randomly allocated across test durations. The adaptive form implemented sampling plans described in García-Pérez and Alcalá-Quintana (2005) and García-Pérez (2014a). For simulations involving the greater–less task, the target number of trials was deployed adaptively via a number of separate staircases implementing the 1-up/1-down rule with steps of the same size as the spacing of test durations under the method of constant stimuli. For simulations involving the same–different task, adaptive deployment implemented the 1-inward/2-outward rule, whereby a “different” response triggers a step in the direction towards the standard, whereas a ”same” response triggers two steps in the direction away from the standard. In either case, half of the staircases started at the longest test duration and the other half started at the shortest test durations. The total number of staircases covaried with the overall number of trials to render an even number of staircases, each running for 10 trials.

Models were fitted to the data generated for each replicate using a fortran version of the software in García-Pérez and Alcalá-Quintana (2017). Parameter estimates (i.e., β and δ for greater–less data or β, δ_1_, and δ_2_ for same–different data) and the *G*^2^ goodness-of-fit statistic, the degrees of freedom, and the *p* value for each replicate were stored for analysis.

### Results

We present detailed results for the subset of conditions with log spacing of 10 test durations. Linear spacing and/or fewer (7, 8, or 9) or greater (11, 12, or 13) test durations did not produce meaningfully different results (see the Discussion section).

**Fig. 6:**
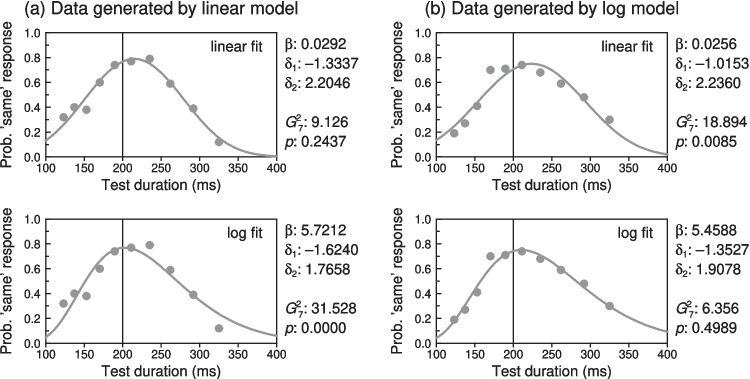
Artificial same–different MOCS data (symbols) generated in 1,000 trials by the linear model with β = 0.03, δ_1_ = −1.4536, and δ_2_ = 2.1101 (left column) or the log model with β = 6 and the same values for δ_1_ and δ_2_ (right column). The top row displays fitted psychometric functions of linear duration (curves) with parameters and fit statistics printed on the right of each panel, where the subscript for *G*^2^ indicates the number of degrees of freedom; the bottom row displays fitted psychometric functions of log duration (curves) with parameters and fit statistics also printed on the right of each panel. The standard duration *x*_s_ = 200 ms is indicated by a vertical line in each panel. The 10 logarithmically spaced test durations are 123, 137, 153, 170, 190, 211, 235, 262, 292, and 325 ms

For a glimpse at the outcomes that we sought to compare, Fig. 6 shows results for one of the replicates in the condition involving the same–different task with *T* = 1,000 trials administered with MOCS when data are generated with the linear model (Fig. 6a) or the log model (Fig. 6b). The top and bottom panels in each column show the results of the linear and the log fit, respectively. With this number of trials, the bilateral symmetry around the standard duration of data generated by the linear model (Fig. 6a) is clearly noticeable by eye, and the path of the data can be very well captured by the symmetric psychometric function of duration (top panel) and not so by the asymmetric psychometric function of log duration (bottom panel). Note also in the values of *G*^2^ and their associated *p* values with seven degrees of freedom that the fitted model is not rejected in the top panel but it is rejected in the bottom panel. In contrast, the bilateral asymmetry of data generated by the log model (Fig. 6b) is also clearly noticeable by eye with this number of trials, and the path of the data can be very well captured by the asymmetric psychometric function of log duration (bottom panel) and not so by the symmetric psychometric function of duration (top panel). Note also that the fitted model is not rejected in the bottom panel and it is rejected in the top panel.

**Fig. 7:**
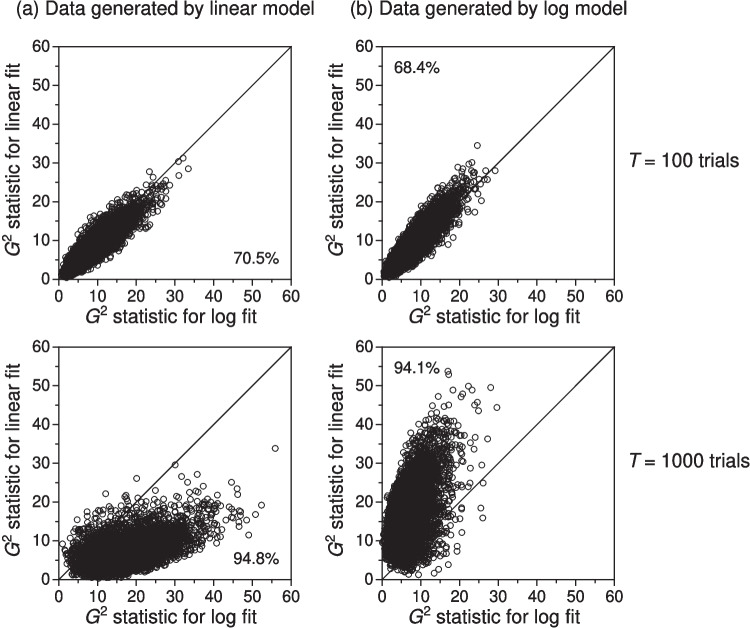
Scatter plot of fit statistics for the linear model against fit statistics for the log model for same–different MOCS data generated over 100 trials (top row) or 1,000 trials (bottom row) under the linear model (left column) or the log model (right column). Results pertain to the condition of 10 logarithmically spaced test durations. Symbols below the diagonal are cases in which the linear model fits better than the log model; symbols above the diagonal are cases in which the log model fits better than the linear model. Inset numerals indicate the percentage of cases in which fit statistics favor the model that generated the data across the 5,000 replicates in each condition

Sample results in Fig. 6 are illustrative but somewhat anecdotal. We are interested in a broader picture of discernibility across the cells of our design. Thus, we first analyzed how often the *G*^2^ statistic for the matched model (i.e., the model that generated the data) attained a smaller value than the *G*^2^ statistic for the non-matched model. We use *G*^2^ and not its *p* value because *G*^2^ is an interpretable measure of discrepancy, and the number of degrees of freedom is the same for both fitted models. Figure 7 illustrates the utility of this analysis for the same–different task with *T* = 100 trials (top row) or *T* = 1,000 trials (bottom row) administered with MOCS. Each symbol in each panel pertains to one of the replicates generated under the linear model (Fig. 7a) or the log model (Fig. 7b), and the scatter plot displays the value of *G*^2^ in the linear fit against the value of *G*^2^ in the log fit for the same data. The inset numerals indicate that identification of the generating model is slightly above chance with 100 trials (top row), whereas identification improves substantially with 1,000 trials (bottom row).

**Fig. 8:**
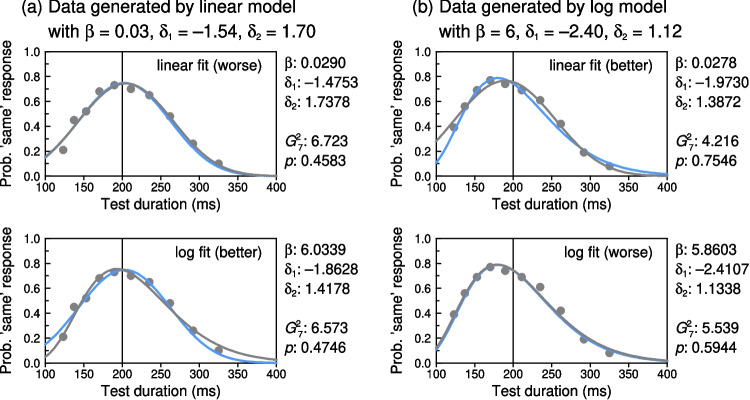
Artificial same–different MOCS data (symbols) generated in 1,000 trials by the linear model (left column) or the log model (right column) with parameters indicated at the top. Graphical conventions as in Fig. 6. The blue curve in each panel is the generating psychometric function

It is nevertheless remarkable that, even with 1,000 trials (bottom row in Fig. 7), the generating model may fit the data worse than the impostor model. This is certainly uncommon (i.e., in less than 6% of the cases in the bottom row of Fig. 7), but it clearly indicates that the clean picture in Fig. 6 is not to be expected across the board. The reason for the unexpected (however infrequent) outcome lies in an unfortunate coupling of parameter values of the generating psychometric function and test durations used to probe it. In other words, the sample values may be such that the impostor psychometric function may fit the data even better than the originating function. This is illustrated in Fig. 8. The fitted (gray curves) and generating (blue curves) functions are virtually identical in the matching case (top panel in Fig. 8a and bottom panel in Fig. 8b), and yet minor quirks in the observed proportions of “same” responses allow the impostor function to accommodate the data better via misestimates of δ_1_ and δ_2_ (compare these misestimates with true values at the top of each column in Fig. 8). This results in a lower value for *G*^2^ in the bottom panel than in the top panel of Fig. 8a and, analogously, in the top panel than in the bottom panel of Fig. 8b.

**Fig. 9:**
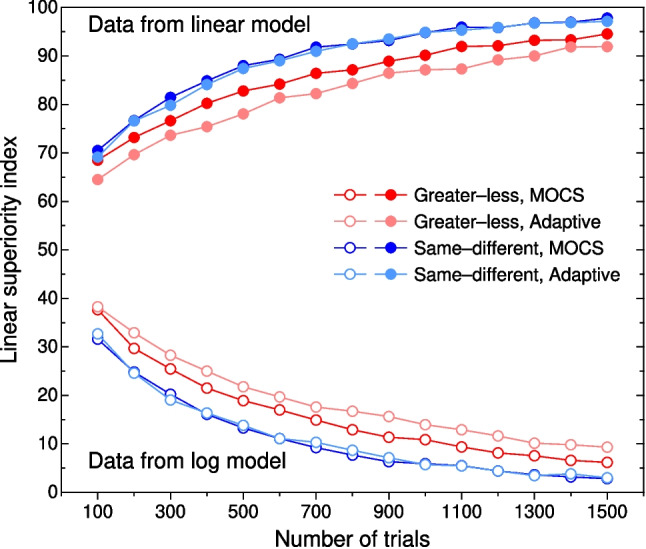
Value of the linear superiority index as a function of number of trials for two different psychophysical tasks (greater–less and same–different) with data collected by MOCS or adaptively (see legend). The top (solid symbols) and bottom (open symbols) strands pertain to data generated by the linear model and by the log model, respectively, with 10 logarithmically spaced test durations

The overall picture of discernibility across simulation conditions (same–different versus greater–less tasks and MOCS versus adaptive placement) as a function of number of trials is displayed in Fig. 9. The ordinate of each symbol represents a linear superiority index defined as the percentage of cases in which the *G*^2^ statistic for the fit of the linear model attained a smaller value than the *G*^2^ statistic for the fit of the log model. Linear superiority indices near 50% indicate indiscernibility of the generating model; in contrast, indices approaching 0% or 100% indicate that the generating model (log or linear) is identifiable in the data. This linear superiority index turns out to be high for data generated by the linear model (solid symbols in Fig. 9), and it is low for data generated by the log model (open symbols in Fig. 9).

The curves described by the strands of symbols in Fig. 9 reveal similar levels of discernibility of both generating models and show some systematic patterns that are identical regardless of which model generated the data. Such systematic patterns comprise (i) a slow but steady increase in the percentage of correct identifications as the number of trials increases, (ii) a uniformly higher percentage of correct identifications from same–different data (strands of blue symbols) compared to greater–less data (strands of red symbols), and (iii) little difference in results for MOCS versus adaptive methods for same–different data (strands of dark blue and pale blue symbols) and a slight but consistent advantage of MOCS over adaptive methods for greater–less data (strands of dark red and pale red symbols).

### Discussion

The goal of our main simulation was to determine the type of task, sampling plan, and number of trials that an empirical study should use to confidently answer the question of whether duration discrimination data from human observers obey psychometric functions of duration or log duration. From the results just presented, the optimal design of an empirical study aimed at answering this question would use the same–different task with trials deployed either via MOCS or adaptively. As for the overall number of trials, *T* = 1,200 seems a good compromise: From Fig. 9, larger numbers of trials do not seem to improve discernibility meaningfully, while they result in much greater burden on observers.

This conclusion holds for the conditions for which detailed results were just presented, which involve a set of 10 test durations (out of conditions in which this set ranged from 7 to 13 durations) that were logarithmically spaced (when linearly spaced test durations were also included in the stimulation study). The results for the remaining conditions did not differ meaningfully, and their presentation is deferred to Part A of the Supplementary Material.

A further characteristic of our main simulation was the use of a single value for β in the psychometric function of all replicates (i.e., β = 0.03 for the linear model and β = 6 for the log model). This was done to avoid losses in degrees of freedom that might differentially affect the models or conditions under comparison, but this decision makes the simulation potentially inadequate for guiding the design of an empirical study aimed at investigating whether actual duration discrimination data conform to psychometric functions of duration or log duration. The decision implies that the set of test durations in any simulation condition involves the same coverage of the psychometric function across replicates. However, in empirical practice, the slope/spread parameter of the psychometric function varies across observers, and thus the selected set of test durations in a study implies a different coverage of the psychometric function of each individual observer. The coverage of the psychometric function might hamper the chances of identifying the generating model beyond the circumstances illustrated in Fig. 8, and thus it may alter the discernibility rates reported in Fig. 9.

**Fig. 10:**
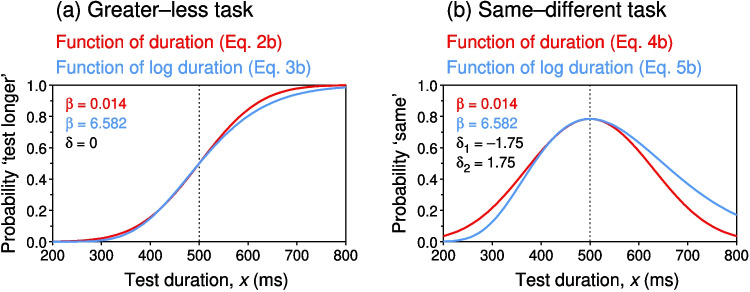
Differences between psychometric functions of duration (red curves) and log duration (blue curves) that are matched in slope in greater–less tasks (left panel) and same–different tasks (right panel). The inset in each panel indicates parameter values in the applicable equations. Unlike in the illustrations of Fig. 8, the values of δ, δ_1_, and δ_2_ do not vary for functions of duration or log duration

The reasons for this concern are illustrated in Fig. 10, which shows differences between psychometric functions of duration and log duration that are matched in slope within and across greater–less and same–different tasks. In the greater–less task (Fig. 10a), meaningful differences exist only within a narrow range of test durations above the standard. In the same–different task (Fig. 10b), differences are comparatively larger and they occur everywhere except within a narrow range around the standard duration. These characteristics are at the base of one of the results of our simulation study, namely, the superiority of the same–different task at distinguishing whether data have been generated by one or the other type of function. At the same time, this illustration shows that the relative location of any selected set of test durations will affect the informative value of the data thus collected. For this reason, we conducted further simulations identical to those reported above except that β varied randomly across replicates, specifically with β uniformly distributed on [0.03 0.06] in the linear case and on [6, 10] in the log case. Part B of the Supplementary Material displays the results, and the good news arising from them is that (i) identifiability of the generating model is not seriously threatened by variability in β, and (ii) the same–different task remains better than the greater–less task as a method to investigate whether data conform to psychometric functions of duration or log duration.

Because variations in β imply variations in the DL, the extra simulations just mentioned allow us to assess the extent to which fitting an inappropriate psychometric function deteriorates estimates of the DL beyond the purely theoretical illustration in Fig. 3 above. We will restrict our illustration to the greater–less task because the definition of the DL for same–different data does not relate to percentage points on the psychometric function (see García-Pérez & Alcalá-Quintana, 2017; see also the appendix in García-Pérez & Alcalá-Quintana, 2020b). Note that defining the DL as the distance (in ms) between the 75% and 50% points on the psychometric functions in Eqs. 2b and 3b yields, respectively,

6a $${\mathrm{DL}}=\frac{\sqrt{2}{\hspace{0.17em}}{\Phi }^{-1}(0.75)}{\beta }$$

6b $$\mathrm{DL}={x}_{s}\hspace{0.17em}\mathrm{e}\mathrm{x}\mathrm{p}\left(\frac{\delta }{\beta }\right)\left(\mathrm{e}\mathrm{x}\mathrm{p}\left(\frac{\sqrt{2}\hspace{0.17em}{\Phi }^{-1}(0.75)}{\beta }\right)-1\right),$$

where Φ^−1^ is the inverse unit-normal cumulative distribution function.

**Fig. 11:**
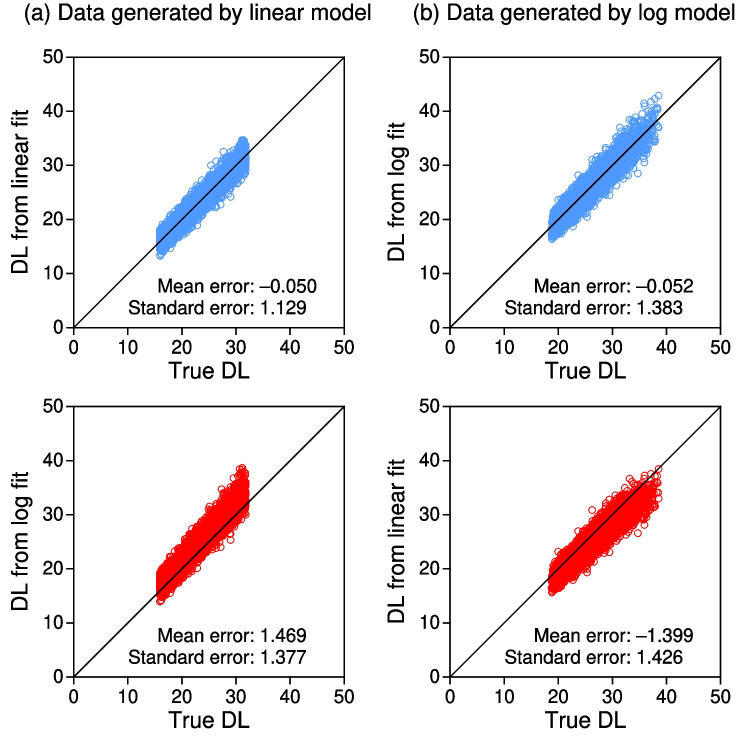
Accuracy of DL estimates for data generated by the linear model (left column) or the log model (right column). Results are shown for the simulation condition with random values for β and data generated with MOCS for 1,200 trials deployed over 10 logarithmically spaced test durations. The top row plots estimated DL against true DL for each of the 5,000 replicates (symbols), with all DLs estimated using the appropriate equation (Eq. 6a in the left panel and Eq. 6b in the right panel). The standard error indicated in each panel is the standard deviation of residuals defined as the difference between estimate and true value. The bottom row plots DLs computed with the wrong equation against true DLs

Before assessing the consequences of estimating DLs with the wrong equation, the top row of Fig. 11 shows scatter plots of proper DL estimates against true DLs for data generated with the linear model (left panel) or the log model (right panel), in both cases for data simulated with 1,200 trials deployed by MOCS over 10 logarithmically spaced test durations. DL estimates are obtained by Eqs. 6a (when data are generated by the linear model) or 6b (when data are generated by the log model) using parameter estimates, whereas true DLs are obtained by the same equations but using generating parameter values. The tight relation in both cases indicates that the use of the appropriate equation makes it possible to estimate the DL without bias and with similar precision in both cases, a precision that also increases as the overall number of trials increases (see the description in the next paragraph). More importantly, the bottom row in Fig. 11 shows that DLs estimated with the equation from the wrong model are biased and have slightly larger standard errors. Specifically, when data are generated by the linear model, Eq. 6b for the log model biases estimates of the DL positively (bottom panel in Fig. 11a). The same holds when data are generated by the log model and Eq. 6a for the linear model is used to estimate the DL, except that the bias is now negative (bottom panel in Fig. 11b).

Figure 11 also illustrates that our simulations allow us to assess accuracy in parameter estimation, a characteristic that can be investigated as a function of all the factors that were manipulated. The picture that arises from our simulations is similar to that reported in earlier studies regarding a reduction of bias and standard errors as the number of trials increases (see, e.g., García-Pérez, 2001, 2014a; García-Pérez & Alcalá-Quintana, 2005, 2020a; Smits & Houtgast, 2006; Smits et al., 2022; Xu et al., 2024). We are deferring the presentation of these tangential results to Part C of the Supplementary Material.

## Empirical study

This section reports the results of a study that collected duration discrimination data from human observers. As in the simulation study described in the preceding section, psychometric functions of duration and log duration were fitted to the data from each observer, and the resultant *G*^2^ statistics were plotted against each other as in Fig. 7, with the expectation that the location of the symbols (massively above or below the diagonal) reveals which type of function best describes the outcomes of the unknown process that generates data for human observers. Based on the results of our simulation study, a same–different task was used, and data were collected over 1,200 trials deployed with adaptive methods that sampled 11 test durations spaced logarithmically around the standard duration.

The purpose of this empirical study is to determine whether psychometric functions of duration or log duration fit data better, under the sampling plan and design that our simulation study showed should give rise to large and informative differences in goodness of fit (not to be confused with evidence in favor of a given data-generation mechanism). This study does not aim to test statistical hypotheses that require consideration of statistical power. The preceding simulations show that, with a conveniently large number of trials, the linear superiority index leans towards one of its extremes with sufficient clarity to support psychometric functions of one type or the other. Thus, this is a descriptive study without hypothesis testing, and the sample size should simply be sufficiently large to be confident that the estimate of the linear superiority index is not grossly mislocated (i.e., that it does not happen to land on the untrue side). Since our simulation results lead us to expect the estimated linear superiority index to be substantially far from 50%, we reckoned that an analysis of at least 40 psychometric functions would be sufficient for our purposes.

The protocol of the study was approved by the institutional ethics committee (refs. CE_20211118-02_SAL and 436_CE_20250911_08_SOC).

### Observers

Twenty-six observers (6 male) with self-reported normal hearing participated in the study. Their ages ranged from 18 to 65 years (median 18 years), they all signed a consent form prior to participation in the study, and, except for the authors (observers #1 and #2), all were naïve as to the goals of the study. It should be stressed that awareness of goals or even holding a preference for the direction of the results is inconsequential in psychophysical studies, as observers cannot give trial-by-trial responses that will collectively be better captured by a function of some preconceived form.

### Apparatus and stimuli

Temporal durations were delivered binaurally via Sennheiser HD 620S headphones (https://www.sennheiser-hearing.com/en-us/p/hd-620s). The stimulus was a 600-Hz square-wave tone produced by the tone() library (https://docs.arduino.cc/language-reference/en/functions/advanced-io/tone) on an Arduino Nano V3.0 board with ATmega328 MCU (https://www.arduino.cc). Presentation of stimuli was controlled via a National Instruments PCI-6509 digital I/O device (https://www.ni.com/en-us/support/model.pci-6509.html) driven by functions in the MATLAB data acquisition toolbox (https://www.mathworks.com/products/data-acquisition.html). All experimental events were under computer control via custom MATLAB scripts that also called psychtoolbox-3 (http://psychtoolbox.org) functions. Presentation durations were delivered with an error of less than 1 ms via the custom-built pauses function (https://www.mathworks.com/matlabcentral/answers/37716-pause-function-in-matlab-for-1-millisecond#answer_212307), not to be confused with the inaccurate built-in pause function in MATLAB (https://www.mathworks.com/help/matlab/ref/pause.html; see also https://www.mathworks.com/matlabcentral/answers/1887467-why-is-pause-function-so-inaccurate-on-windows#answer_1139927).

Three standard durations *x*_s_ were selected (150, 300, and 450 ms). The 300-ms standard was used with all observers, and the other standards were used according to each observer’s availability. For each standard duration, 11 test durations were defined that ranged from ${x}_{s}\times {2}^{-0.5}$ ms to ${x}_{s}\times {2}^{0.5}$ ms in equally spaced binary log steps (i.e., the exponent of 2 ranges from −0.5 to 0.5 in steps of 0.1). All test durations were rounded to the nearest millisecond.

### Procedure

Testing took place in a quiet room under standard office lighting. Observers sat comfortably wearing headphones and with easy access to the response interface (a numeric keypad).

A dual-presentation task with the same–different response format was used. Collection of data for a given standard was planned to take place in six 200-trial blocks separated by short breaks at each observer’s discretion, but some observers completed only five blocks. Immediately before the first block, observers took between 32 and 64 practice trials until they gained familiarity with the perceptual judgment and the response interface. A trial consisted of presentation of the standard duration followed by presentation of a selected test duration.^2^ The inter-stimulus interval was 800 ms for the 150-ms and 300-ms standards and 1,000 ms for the 450-ms standard. Observers had unlimited time to respond whether the tones appeared to have the same duration (by pressing the “5” key on the numeric keypad) or the tones appeared to differ in duration (by pressing the ”2” key on the numeric keypad). An extra key (”0” on the keypad) was enabled for observers to ask for the trial to be rescheduled if they had had a lapse of attention or they had missed the presentations for whatever reason. Observers were warned that the trial would not be repeated immediately afterwards and that they should not use this key to give themselves a second chance with the same pair. Unbeknownst to observers, the response on any trial was also discarded and the trial rescheduled if the nominal and actual presentation duration of any of the tones differed by 2.5 ms or more. Actual presentation duration was estimated with a MATLAB tic–toc sequence bracketing calls to the functions switching the tone on and off.^3^ Trial administration within each block was self-paced, as the next trial started 500 ms after a response to the current trial had been entered. Across observers and standard durations, the minimum duration of a block of 200 trials ranged from 9.1 min to 16.6 min, with a median of 11.8 min, and the maximum duration ranged from 10.2 min to 23.3 min with a median of 13.8 min. The aggregated duration of all blocks (excluding self-administered breaks) for any given standard duration ranged across observers from 56.6 min to 113.7 min, with a median of 74.6 min.

The choice of test duration for each of the 200 trials in each block was governed by 10 interwoven adaptive staircases, each completing 20 trials. Half of the staircases started at the shortest test duration, and the other half started at the longest test duration. The size of the staircase steps was the spacing of durations defined earlier. All staircases implemented the 1-inward/2-outward rule by which a “different” response sets the next test duration one step closer to the standard duration, whereas a “same” response sets the next test duration two steps away from it in the direction in which the staircase is currently moving (García-Pérez, 2014a).

### Results and discussion

Because some observers completed sessions for several standard durations and for both orders of presentation of test and standard, we collected data for a total of 45 psychometric functions across the 26 observers. Psychometric functions in Eqs. 4 and 5 were fitted to data aggregated across blocks but separately for each standard duration, order of presentation of standard and test, and observer. We used the same custom software described above for simulated data.

**Fig. 12:**
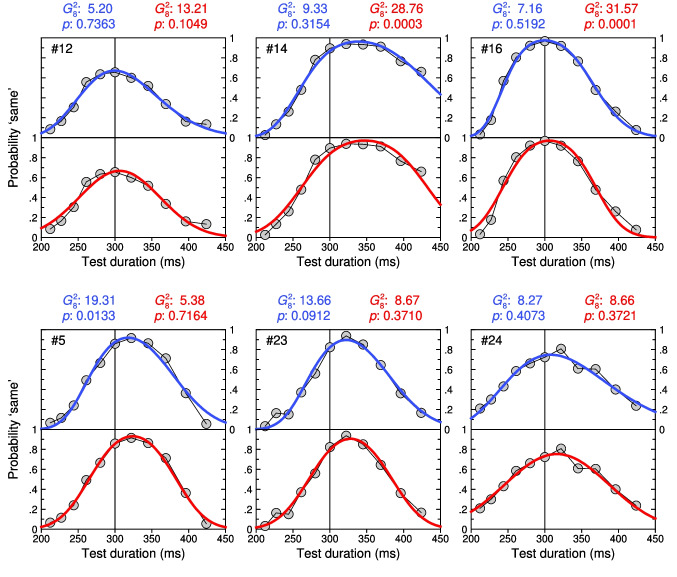
Sample data (symbols) and fitted psychometric functions of duration (red curves) or log duration (blue curves) for six observers with a standard duration of 300 ms (vertical dashed line in each panel). Vertically abutted panels display the same data and differ only in the type of psychometric function fitted to them. Data points are connected to facilitate visual comparison of the path followed by the data and the path of the fitted curve. Goodness-of-fit statistics and *p* values are printed for linear and log fits at the top of each pair of abutted panels. The upper part shows cases in which the log fit is clearly better than the linear fit; the lower part shows two cases in which the linear fit is better (leftmost and center pair of panels) and one in which linear and log fits are indistinguishable (rightmost panels)

Sample data and fitted psychometric functions are shown in Fig. 12. The top half displays selected cases in which the log fit (upper panels, in blue) is better than the linear fit (lower panels, in red), even by eye; the bottom half displays selected cases in which the linear fit appears better or in which linear and log fits appear similar. Part D of the Supplementary Material shows all cases in graphical form and includes a table listing parameter estimates and measures of fit under each model for each case. More often than not, the log fit was superior to the linear fit (as in the top half of Fig. 12), both by the *G*^2^ statistic and in a visual comparison of the path described by the data (lines connecting data points in each panel) and the path described by the fitted function (red or blue curve in each panel). Cases in which the linear fit was superior to the log fit (as in the leftmost panel in the bottom half of Fig. 12) were scarce. When visual inspection did not support one type of psychometric function more than the other type (as in the center and rightmost panels in the bottom half of Fig. 11), the *G*^2^ statistic generally favored the log fit.

**Fig. 13:**
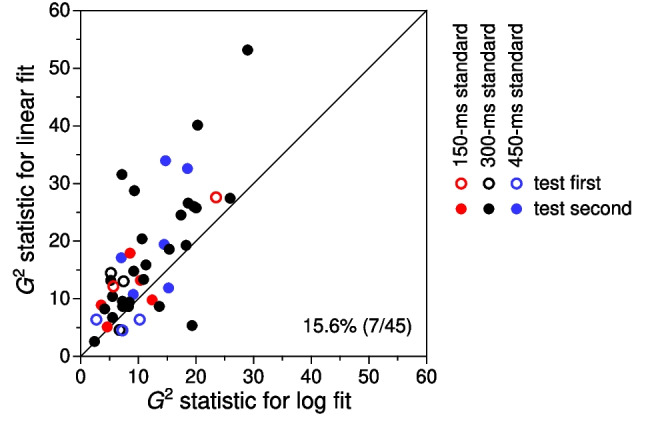
Scatter plot of values of *G*^2^ from the linear fit (vertical axis) against values of *G*^2^ from the log fit (horizontal axis) of the same data. Each individual symbol comes from a different case in the condition indicated by the type and color of the symbol (see the legend on the right). The diagonal identity line is plotted for reference. The inset numerals at the bottom right indicate the linear superiority index (15.6%), the number of cases (7) in which the value of *G*^2^ from the log fit is larger than the value of *G*^2^ from the linear fit, and the total number of cases (45). Graphically, the linear superiority index is the percentage of cases that lie below the diagonal

For a summary description, Fig. 13 shows a scatter plot of measures of fit in the form of Fig. 7, with the linear superiority index printed at the bottom-right corner of the panel. Each symbol comes from one of the 45 cases (psychometric functions) collected across observers, standard durations, and orders of presentation of test and standard in each trial (see the legend on the right of Fig. 13). Clearly, the linear fit is inferior to the log fit across the board, and the scatter of data is similar to that displayed by data simulated under the log model for a similar number of trials per psychometric function (compare with the bottom panel in Fig. 7b). Specifically, data points below the diagonal occur only at low values of *G*^2^, and values above the diagonal extend further above and to the right. Thus, even considering that the anomalies discussed with simulated data in Fig. 8 may also occur here, the overall pattern in our empirical data takes the form of data generated by the log model and supports the notion that empirical data are best described by psychometric functions of log duration.

It is noteworthy that the linear superiority index is 15.6% in Fig. 13, which is less extreme than the expectation (which is ~5%) created by our simulation results when data are generated by the log model in 1,200 trials. The reason may lie in true parameter values whose variability and range across observers is larger than those considered in our simulations, at least judging by the variability and range of estimated parameter values in the linear and log fits of empirical data (see the table of parameter estimates in Part D of the Supplementary Material). If the fixed sampling plan used in the empirical study does not cover the observers’ true psychometric functions in the same form as in our simulations, distinguishability may fail to reach the rates expected from simulations. Some indication that this may be the case arises by inspection of the table of parameter estimates, where estimated values for β, δ_1_, and δ_2_ outside the ranges used in simulations are not uncommon.

**Fig. 14:**
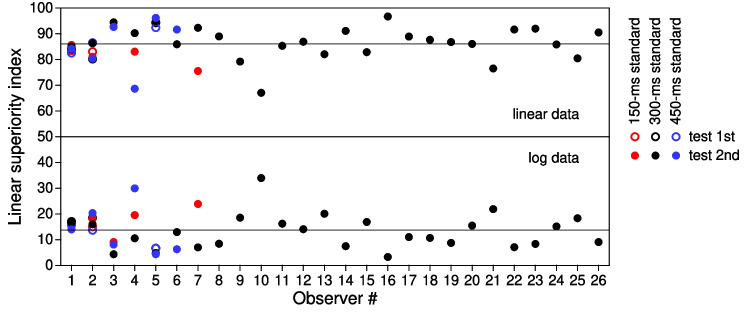
Bootstrap linear superiority indices obtained from the fitted psychometric function for each observer (location along the horizontal axis) and condition (type of symbol; see the legend on the right). Indices computed from data generated with the linear (alternatively, log) model fit turned up above (alternatively, below) the horizontal line at 50%. The average linear superiority index for data generated from the log model (lower dashed horizontal line) is similar to the value obtained with our empirical data (see Fig. 13) and worse than that obtained from our earlier “pristine” simulations

To test the conjecture that mismatch with simulation conditions is responsible for this discrepancy, we used bootstrap methods to conduct a simulation whose setup matched these empirical conditions. Specifically, for each of the 45 cases (combinations of observer, standard duration, and presentation order), we used estimated parameters of the linear fit and the log fit (in separate runs) to generate 2,000 replicates under the exact same empirical conditions of each individual case (number and placement of test durations and overall number of trials). We computed the linear superiority index across all replicates for each case and type of data (i.e., generated by the log model or by the linear model), and we also computed an overall linear superiority index across cases for each type of data. For each individual case, a linear superiority index higher than 5% when data were generated with the log model or lower than 95% when data were generated with the linear model would indicate that the fixed sampling plan and number of trials in our empirical study was not optimally suited to identifying the generating model when psychometric functions had the set of parameter values estimated in our empirical study. The results are shown in Fig. 14.

Each data point pertains to the observer at the abscissa and the condition indicated by the type of symbol (see the legend on the right). Linear superiority indices obtained by simulating the linear or the log models are unmarked, but they turned up located above or below the horizontal line at 50%, respectively, as expected. Note that there are cases in which our common sampling plan renders linear superiority indices away from expectations based on our initial simulations. However, the average linear superiority index across the board lies near 15% for log data (lower dashed horizontal line in Fig. 14). These results concur with the percentage in Fig. 13 and lend support to the notion that empirical duration discrimination data are better described by psychometric functions of log duration.

## Analysis of existing datasets

Numerous duration discrimination datasets from published papers are available in online repositories. These data reflect as many within- or between-subject conditions as necessary to address the topic under study in each paper. They are all suitable for the type of analysis we have conducted here, namely, to check out whether psychometric functions of duration or log duration fit the data better. The number of trials per psychometric function in these studies was as small as the authors judged reasonable given their purposes and circumstances (e.g., the number of within-subject conditions that each observer had to go through). In general, these numbers of trials are far fewer than our simulations showed as necessary for a clear picture such as that obtained in our empirical study. Yet we found no reason to refrain from conducting this analysis, and we expected the results to give some indication (however weak) of which type of psychometric function fit the data better.

A list of potential candidate papers was obtained in a Google Scholar search that looked for the terms “psychometric function” and “duration discrimination” anywhere within documents published in 2017 or later. The 300+ papers thus identified were screened for appropriateness, and the final list of suitable papers was expanded by looking at seemingly suitable papers referenced by the papers in the initial list and by looking at earlier papers published by the authors of papers in the initial list. We downloaded dual-presentation data that had been collected with greater–less or same–different tasks from the online repositories mentioned in 49 of these papers. We also obtained data not available in a repository from the authors of five other papers that included a data availability statement^4^ and from the authors of yet another eight papers that did not include a data availability statement.

Data collected with the greater–less task were fitted with the logistic functions in Eqs. 1, whether the test duration was presented first or second in each trial (but still separately for each presentation order when both had been used); data collected with the same–different task were fitted with the model functions in Eqs. 4 and 5 using the appropriate form according to whether test durations were presented first or second in each trial (and separately for each order of presentation when both had been used). We also included in our analysis data collected with the oddball paradigm if the originating study (or the applicable condition in the study) presented the oddball duration last in the sequence and the task was to judge its duration in comparison to that of the preceding presentations in the same trial. Formally, these data reflect duration judgments analogous to those obtained in classical dual-presentation tasks in which the test duration is presented second, with the only difference that in these cases the standard duration is presented more than once in each trial.

We excluded eight papers that used dual-presentation methods with randomization of the order of presentation of test and standard durations in each trial but whose associated data files did not identify the order of presentation in each trial. The well-documented presence of order effects in dual-presentation methods makes data aggregated across presentation orders uninterpretable (see García-Pérez & Alcalá-Quintana, 2011b, 2019; Ulrich & Vorberg, 2009). More importantly in the present context, the psychometric function for aggregated data is a weighted average of the psychometric functions that hold separately for each order of presentation, and thus, whether these averages are better approximated by functions of duration or log duration is anecdotal and irrelevant to our purposes.

We also downloaded (or requested) data from 15 papers that had used single-presentation methods such as the modified bisection task (Grondin and Rammsayer, 2003) and the temporal generalization task (Wearden, 1992). In these variants of the greater–less and same–different tasks, the standard duration is not presented on every single trial but only repeatedly before the session starts (and perhaps also at intermediate points between blocks of trials within a session) so that the observer memorizes the standard duration and compares the test duration in each trial with the memory of the standard duration. On each trial, the observer responds whether the test duration was equal to or different from the standard duration (temporal generalization task) or whether the test duration was longer or shorter than the standard duration (modified bisection task).^5^ Thus, modified bisection tasks and temporal generalization tasks differ from one another only in the question that observers are asked to answer in each trial, just as it is in the dual-presentation greater–less task compared to the dual-presentation same–different task. Single-presentation methods are inferior to dual-presentation methods in several respects (see García-Pérez, 2014b; García-Pérez & Alcalá-Quintana, 2013, 2019), but their deficiencies are irrelevant to our purposes: We do not plan to make inferences about parameters of the fitted psychometric functions (which is where the problem lies) but only to check out whether functions of duration or log duration describe the data better. Data collected with the modified bisection task were fitted with the logistic functions in Eqs. 1, and data collected with the temporal generalization task were fitted with the model functions in Eqs. 4b and 5b (for nominally test-second presentations, given that the test duration is compared to a standard duration presented earlier).^6^

Altogether, we analyzed data from 69 papers, totaling 17,784 individual psychometric functions across studies, observers, and conditions. Of these, 14,811 psychometric functions came from greater–less tasks, 192 came from same–different tasks, 1,401 came from modified bisection tasks, and 1,380 came from temporal generalization tasks. The number of test durations per psychometric function ranged from 2 to 49, with a median of 7. The number of trials per psychometric function ranged from 21 to 660 with a median of 80, but 92.10% of the psychometric functions were fitted to data from 200 or fewer trials, and 58.53% were fitted to data from 100 or fewer trials. Other characteristics of the studies reported in each paper are described in Part E of the Supplementary Material, which also shows plots of data and fitted psychometric function for each case in each paper.

**Fig. 15:**
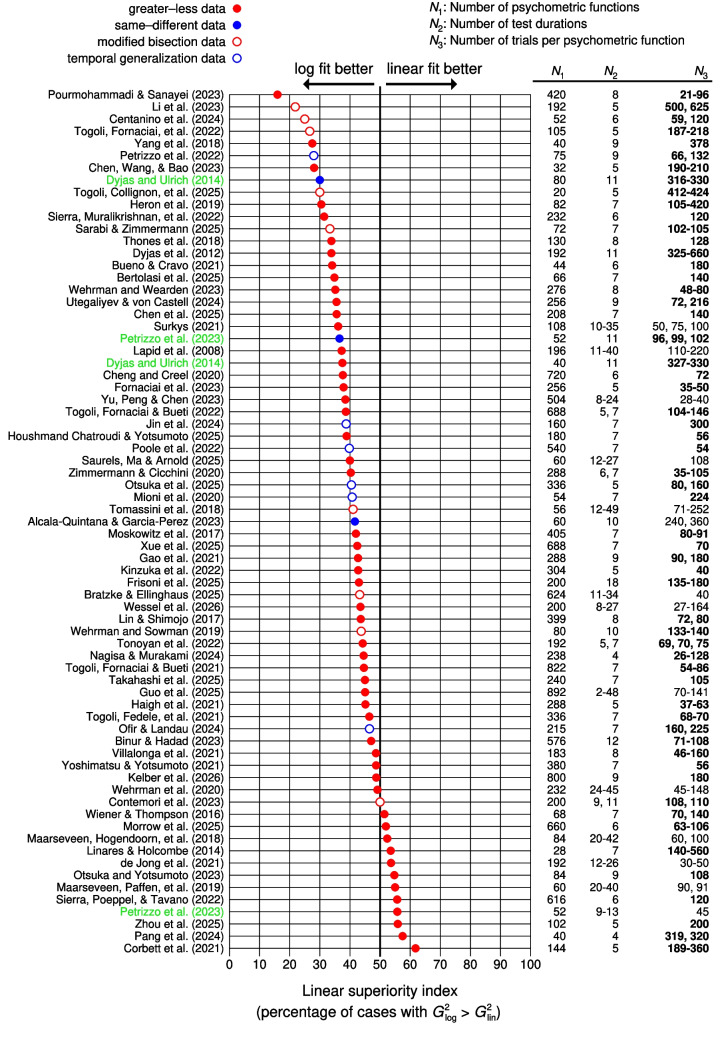
Summary results of the analysis of data from 69 papers. Papers are listed from lowest (top) to highest (bottom) value of the linear superiority index. References in green font appear twice in the list because they reported results for different types of data. Different symbols indicate which type of data was involved in each case (see the legend at the top left). The right columns indicate characteristics of the data coming from each study (see the legend at the top right). Data had been collected with MOCS when *N*_3_ is in boldface; in other cases, data had been collected adaptively or in combination with MOCS.

Figure 15 summarizes the results of our analyses in compact form. Papers are listed downwards^7^ by increasing value of the linear superiority index. The index was computed across all the psychometric functions involved in the paper, whose total is indicated in the column labeled* N*_1_ on the right of Fig. 15. The columns labeled *N*_2_ and *N*_3_ on the right in Fig. 15 indicate the number (or numbers) of test durations per psychometric function and the overall number (or numbers) of trials per psychometric function, respectively. Recall that a linear superiority index above 50% indicates that the linear model fits better across the board, whereas a value below 50% indicates that the log model fits better across the board. In line with the results of our own empirical study, these results also favor the log model although relatively weakly because analyses with small numbers of trials per psychometric function are not conclusive.

## Discussion and conclusion

Our query about the correct type of psychometric function to fit to duration discrimination data started with consideration of Weber’s law, empirical characteristics of data, and theoretical properties of psychometric functions of duration or log duration. Next, we conducted a simulation study to elucidate the amount of data and sampling plan that were best suited to allow the appropriate function to fit better than the other function. These simulations showed that collecting same–different data adaptively across 1,200 trials over 11 test durations logarithmically spaced around the standard duration would result in the generating psychometric function fitting the data better than the impostor function on more than 90% of occasions. This outcome creates a rough expectation for the results of an empirical study, provided that actual psychometric functions are covered similarly with this sampling plan.

Our subsequent empirical study collected data for 45 psychometric functions and found that functions of duration fitted the data better for only seven (15.6%) of them. Additional simulations using parameters of the fitted functions (which were often outside the range of the original simulations) confirmed that this figure is to be expected under the circumstances.

We also conducted analogous analyses of available duration discrimination data from 69 published papers. The number of trials per psychometric function were much lower (median of 80), but the sheer total of 17,000+ psychometric functions across papers and conditions provided naturally weak support to psychometric functions of log duration.

We limited our analysis to logistic functions for greater–less data (Eq. 1) and difference-of-cumulative-Gaussians (Eqs. 4 and 5) for same–different data. We did not ask whether these particular mathematical forms are superior to others (e.g., Weibull, Quick, hyperbolic tangent, or cumulative Gaussian functions; see Strasburger, 2001) because they are all virtually indistinguishable (see, e.g., figure 1 in Ulrich & Miller, 2004). At the same time, they all share distinctively different characteristics when expressed as functions of duration or functions of log duration. We thus have no reason to think that use of other mathematical forms might have favored psychometric functions of duration rather than log duration.

Our empirical results support the notion that duration discrimination data should be fitted with psychometric functions of log duration. Researchers do not have to collect data over as many trials as we did in our study to check that this is true for their own data; they need only fit functions of log duration to their data on grounds of the evidence provided in this paper.

## Data Availability

Data collected in this study are available at https://osf.io/qkugy.

## Footnotes

1 This feature is more conspicuous when the vertical axis represents “proportion correct” so that the data display an asymmetric V shape in which the left wing is steeper than the right wing (see, e.g., figures 7 and 9 in Wehrman & Wearden, 2023).

2 Three observers completed subsequent sessions with additional blocks that were identical in design except that the standard duration was presented second in every trial. These results are also presented here.

3 The validity of such estimates of presentation duration was established in advance by checking them against the actual duration of the electrical signal reaching the headphones.

4 The authors of 23 papers with a statement indicating that data could be obtained from the corresponding author (or with data reportedly deposited in a repository that we could not access) did not respond to an email sent to their current addresses.

5 In the classical bisection task, two extreme (short and long) durations are displayed repeatedly during the training phase. Subsequently, in each trial of the testing phase, observers judge whether the test duration was closer to the short or to the long exemplars. This implies that the observer must keep two durations in memory (the short and the long), in contrast to the modified bisection task in which the observer only needs to memorize one duration (the standard). We decided against including classical bisection data in our analysis because, technically, the observer compares the test duration in each trial with two referent durations, not with the standard duration. Thus, our analysis includes tasks in which the test duration is compared with the standard duration displayed in the same trial (as in the dual-presentation greater–less or same–different tasks) or with a standard duration retrieved from memory (as in the modified bisection or temporal generalization tasks). We must note that the term “modified bisection task” has also been used to denote other variants of the classical bisection task (see, e.g., Poole, 2014; Corcoran et al., 2018; Quan et al., 2025).

6 Arguably, the square-root-of-2 divisor in Eqs. 4 and 5 should be removed when the referent is a single-presentation method because the decision variable in these cases is not the difference between two random variables (see García-Pérez & Alcalá-Quintana, 2013). Note, however, that this is relevant only in the context of comparisons of model parameters across methods in within-subject studies (see, e.g., García-Pérez & Peli, 2014, 2015, 2019). In the present case, the presence or absence of this divisor does not affect the potential fit of the psychometric function because of the inherent unidentifiability (i.e., any arbitrary value for this divisor accompanied by suitable changes in the estimated values of β, δ_1_, and δ_2_ produces the exact same psychometric function).

7 Note that Dyjas and Ulrich (2014) and Petrizzo et al. (2023) are listed twice in Fig. 15 (highlighted in green). This is because both papers included data collected with the greater–less task and data collected with the same–different task, which we consider separately in this analysis (see the color code at the top left of Fig. 15).

## Code availability

MATLAB code used to collect data is available at https://osf.io/qkugy.

## Declarations

### Open practices statement

Original data and supplementary materials for this study are available at https://osf.io/qkugy. The study was not preregistered.
